# Epigenetic Control of Autophagy in Cancer Cells: A Key Process for Cancer-Related Phenotypes

**DOI:** 10.3390/cells8121656

**Published:** 2019-12-17

**Authors:** Paul Peixoto, Céline Grandvallet, Jean-Paul Feugeas, Michaël Guittaut, Eric Hervouet

**Affiliations:** 1 Univ. Bourgogne Franche-Comté, INSERM, EFS BFC, UMR1098, Interactions Hôte-Greffon-Tumeur/Ingénierie Cellulaire et Génique, F-25000 Besançon, France; paul.peixoto@univ-fcomte.fr (P.P.); cgrandvallet25@gmail.com (C.G.); jean-paul.feugeas@univ-fcomte.fr (J.-P.F.); michael.guittaut@univ-fcomte.fr (M.G.); 2 EPIGENEXP platform, Univ. Bourgogne Franche-Comté, F-25000 Besançon, France; 3 DImaCell platform, Univ. Bourgogne Franche-Comté, F-25000 Besançon, France

**Keywords:** autophagy, epigenetics, cancer, DNA methylation, histone deacetylase (HDAC), histone methylation, histone methylation

## Abstract

Although autophagy is a well-known and extensively described cell pathway, numerous studies have been recently interested in studying the importance of its regulation at different molecular levels, including the translational and post-translational levels. Therefore, this review focuses on the links between autophagy and epigenetics in cancer and summarizes the. following: (i) how *ATG* genes are regulated by epigenetics, including DNA methylation and post-translational histone modifications; (ii) how epidrugs are able to modulate autophagy in cancer and to alter cancer-related phenotypes (proliferation, migration, invasion, tumorigenesis, etc.) and; (iii) how epigenetic enzymes can also regulate autophagy at the protein level. One noteable observation was that researchers most often reported conclusions about the regulation of the autophagy flux, following the use of epidrugs, based only on the analysis of LC3B-II form in treated cells. However, it is now widely accepted that an increase in LC3B-II form could be the consequence of an induction of the autophagy flux, as well as a block in the autophagosome-lysosome fusion. Therefore, in our review, all the published results describing a link between epidrugs and autophagy were systematically reanalyzed to determine whether autophagy flux was indeed increased, or inhibited, following the use of these potentially new interesting treatments targeting the autophagy process. Altogether, these recent data strongly support the idea that the determination of autophagy status could be crucial for future anticancer therapies. Indeed, the use of a combination of epidrugs and autophagy inhibitors could be beneficial for some cancer patients, whereas, in other cases, an increase of autophagy, which is frequently observed following the use of epidrugs, could lead to increased autophagy cell death.

## 1. Introduction

### 1.1. Basics of Autophagy

**Figure 1:**
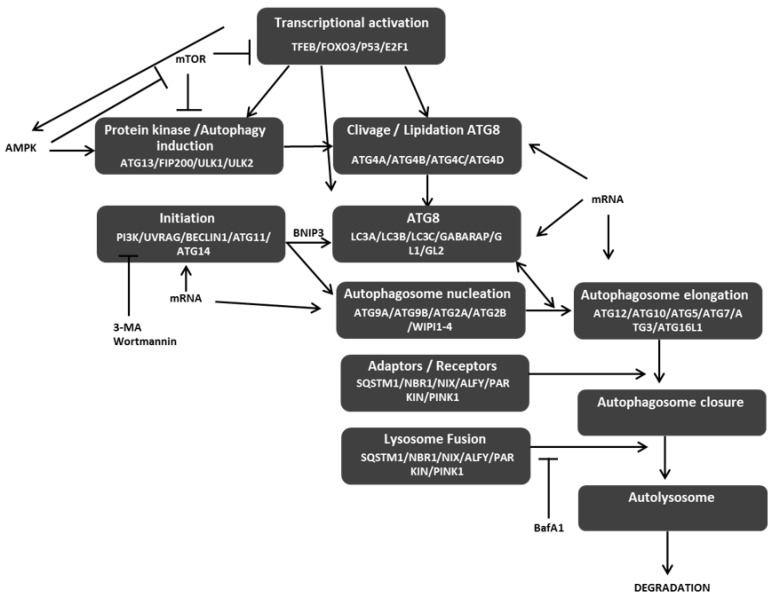
Interconnection of major autophagy steps. Autophagy induction and initiation of autophagy steps lead both to the cleavage and lipidation of autophagy gene 8 (ATG8) and autophagosome nucleation. ATG8 and protein involved in autophagosome elongation favor the closure of autophagosome and, then, its fusion with lysosome to form an autolysosome and to induce the degradation of its content. AMPK acts as an activator of autophagy, whereas mTOR acts as an inhibitor of autophagy. 3-MA and wortmannin are chemical inhibitors of early steps autophagy, whereas BafA1 blocks the fusion of autophagosomes with lysosomes. Arrows represent a positive action on the target, whereas bar-headed arrows represent an inhibition process.

Autophagy is a multistep process involving more than 40 autophagy-related (ATG) proteins leading to the formation of a double membrane structure, called autophagosome, and the elimination of its content following its fusion with a lysosome (Figure 1) [1]. Autophagy degrades proteins or organelles, such as damaged mitochondria, and is frequently linked to cancer initiation and progression. Indeed, both pro- (e.g., resistance to starvation, chemo-resistance, etc.) and anticancer (e.g., autophagic cell death. etc.) properties have been associated to autophagy depending of the cell model, cancer grade, or cell microenvironment. Autophagy can be divided into the following four steps: (i) initiation which is controlled by the activating phosphorylation of ULK1 (Unc-51-like autophagy activating kinase 1) by AMPK while the phosphorylation of ULK1/ULK2 by mTOR (mammalian target of rapamycin kinase) leads to autophagy inhibition; (ii) formation of the phagophore which requires Beclin-1 and ATG14 (autophagy gene 14) leading to the recruitment of the PI3K kinase and the production of PI3P, shown to be essential for the phagophore initiation; (iii) elongation which is dependent of two complexes, the ATG5/ATG12/ATG16L trimeric complex which acts as an E3-like molecule and facilitates the conjugation of ATG8 proteins onto phospholipids, previously cleaved by the ATG4 proteases; and (iv) fusion involving UVRAG (ultraviolent irradiation resistance-associated), and leading to the fusion of the autophagosome with lysosomes to induce the degradation of its content by proteolytic lysosomal enzymes. Autophagy can be unselective, when the phagophore nonspecifically engulfs part of the cytoplasm, or selective, when adaptor proteins specifically link the material to degrade and the ATG8 proteins on the surface of the autophagosome to induce its selective degradation. We can cite, for example, the mitophagy, a process implying the protein NIX which specifically targets mitochondria to autophagosomes thanks to its interaction with ATG proteins. 

### 1.2. Transcriptional Regulation of Autophagy

Although autophagy is a well-known and extensively described cell pathway, numerous studies have been recently interested in studying the importance of its regulation at different molecular levels, including at the translational or post-translational levels. Indeed, only a few transcriptional factors (TFs) have been described to be key regulators of the transcription of autophagy genes (for reviews see [2,3]). The helix–loop–helix transcription factor EB (TFEB) can preferentially bind to the coordinated lysosomal expression and regulation motif (CLEAR) (GTCACGTGAC) and promote the transcription of many ATG-related genes. Indeed, upon starvation, TFEB is dephosphorylated and translocated into the nucleus leading to an increase in autophagy gene transcription, and therefore autophagy. Under nutrient-rich conditions, mTOR is responsible for the phosphorylation of TFEB at the lysosomal surface, and therefore leading to its inactivation. Phosphorylation of the forkhead transcription factor FOXO3 by AKT blocks its translocation into the nucleus, therefore, inhibiting the transcription of several ATG-related genes. Another transcription factor, P53, which is frequently mutated in cancer cells, also promotes the expression of FOXO3. Moreover, P53 can also induce the expression of Sestrin proteins which activate AMPK leading to the inhibition of mTOR and the activation of autophagy. Finally, hypoxia, which could be observed in solid tumors, is associated with a decreased recruitment of NFKB and E2F1 on the *BNIP3* promoter and an inhibition of the transcription of *BNIP3*. These data, therefore, strongly argue that autophagy can, indeed, be controlled at the transcription level. 

This review focuses on the links between autophagy and epigenetics in cancer to describe the following: (i) how *ATG* genes are regulated by epigenetics, including DNA methylation and post-translational histone modifications; (ii) how epidrugs are able to modulate autophagy in cancer and to alter cancer-related phenotypes (proliferation, migration, invasion, tumorigenesis, etc.) and; (iii) how epigenetic enzymes can also regulate autophagy at the protein level. One noteable observation was that researchers most often reported conclusions about regulation of the autophagy flux by epigenetic modifications or epidrugs, by only analyzing the levels of the LC3B-II form in treated cells. However, it is now widely accepted that an increase in the LC3-II form could be the consequence of an induction of the autophagy flux, as well as a block in the autophagosome-lysosome fusion and therefore vesicle degradation. We systematically reanalyzed all the published results describing the link between epidrugs and autophagy to determine whether autophagy flux was indeed regulated by epidrugs. To do so, we determined whether the conclusions of the authors were based on different protocols analyzing autophagy flux following a treatment with an epidrug (LC3B-II levels, number of autophagosomes in presence and absence of inhibitors of autophagy induction, and autophagosome-lysosome fusion, etc.) or whether the conclusions were only based on the analysis of the LC3B-II levels. 

Therefore, to the best of our knowledge, this review summarizes, for the first time, the recent data describing a new approach to regulate autophagy during the development of cancers. These data clearly demonstrate that some cancer cells could profit from the use of a combination of epidrugs and autophagy inhibitors while, in other cancers, an increase of autophagy, which is frequently observed following the use of epidrugs, led to increased autophagy cell death.

## 2. Regulation of Autophagy Genes in Cancer Cells by DNA Methylation

Epigenetics is a transmissible but reversible process controlling gene expression. Among epigenetic modifications occurring in promoters, DNA methylation is a mark affecting DNA, whereas histone post-translational modifications modify the chromatin. DNA methylation and histone modifications both regulate gene transcription by modulating local chromatin structure and selective fixation of chromatin readers.

### 2.1. Basics of DNA Methylation

DNA methylation is the process leading to the addition of a methyl group onto the fifth carbon of a cytosine located in CpG motifs. About 80% of CpGs in the genome are methylated in mammals and this epigenetic mark is generally associated to gene repression and heterochromatin condensation. DNA methylation is catalyzed by a family of enzymes, called the DNA methyl transferases (DNMTs). On the one hand, DNMT1 mainly regulates the maintainance of DNA methylation on the newly synthetized DNA strand following DNA replication using the parental methylated strand as a matrix. DNMT3A and DNMT3B, on the other hand, are involved in de novo methylation on both stands of DNA, a process which is independent of the S-phase replication, and their roles during embryogenesis and inactivation of tumor suppressor genes (TSG) in cancers are well described. Another enzyme, DNMT3L, does not contain any catalytic domain but has been shown to be able to activate the latter enzymes. DNA methylation has been closely associated to tumorigenesis. For example, a global DNA hypomethylation is frequently observed in tumors and is correlated to grade. Local hypomethylation, as well as local hypermethylation, could also, respectively, lead to the expression of specific genes (e.g., oncogenes, antiapoptotic genes, etc.) or the specific inhibition of gene expressions (TSG, proapoptotic genes, etc.) (for a review, [4]). 

### 2.2. Negative Regulation of Autophagy by DNA Methylation Favors Cancer Cell Aggressiveness

#### 2.2.1. Molecular Mechanisms Inhibiting ATG Gene Expression via DNA Methylation 

ChIPsequencing analyses showed in a prostate cancer cell model that the co-repressor DAXX (death domain associated protein) colocalized with DNMT1 near the TSS (transcriptional start site) of several ATG-related genes including *ULK1* and *DAPK3* (death-associated protein kinase 3) [5]. Since DAXX has already been shown to specifically recruit DNMTs on specific target promoters, these data strongly suggested that DAXX may actively mediate a methylation-dependent silencing of *ATG* genes [6]. Moreover, it has been shown that DNMT1 expression levels are strongly increased in childhood acute lymphatic leukemia (ALL) as compared with healthy individual samples. DNA digestion of ALL samples using restriction enzymes able to target modified CG sites showed a protection of digestion of *ATG5* and *LC3B* promoters confirming CG methylation; it has been shown that the promoter methylation of these genes is correlated with decreased expression levels [7]. Interestingly, these publications also demonstrated that DNA methylation-dependent inhibition of ATG-related gene expression, in many different cancer models, led to the promotion of aggressiveness by reducing autophagy cell death. 

#### 2.2.2. Negative Regulation of ATG Genes by DNA Methylation in Cancer Cells

Several genes directly involved in the core of the autophagy process have been shown to be controlled by DNA methylation in cancers (these genes are listed in the Table 1). Indeed, the expression of *BECN1* (Beclin 1) is frequently inactivated by a loss of heterozygosity (LOH) combined with a promoter hypermethylation in invasive breast cancers [8]. Similarly, *ATG4D*, *ATG2B*, *ATG9A*, and *ATG9B* promoter hypermethylation have been found in most of invasive ductal carcinomas (IDC) and this increased methylation was correlated to the grade of cancer, a decreased gene expression, and lymph node infiltration [9]. Hypermethylation of *BNIP3* promoter (BCL2/adenovirus E1B 19 kDa protein-interacting protein 3) was also reported in colorectal carcinomas and a 5-aza-deoxycytidine (5-azadC) treatment restored its expression in colorectal cancer cells [10]. Similarly, combined 5-aza-dC and TSA (trichostatin A) treatments restored *GL1* (GABARAPL1, GABA type A receptor-associated protein like 1) expression in breast cancer cell models [11]. In both gastric carcinoma and non-cancerous mucosae, methylation of the promoter of *MAP1LC3v1* (microtubule-associated protein 1 light chain 3 alpha variant 1, also called LC3B) decreased its expression and was associated with *Helicobacter pilori* infection [12]. Since the inhibition of *MAP1LC3v1* expression, in gastric cancer cells, favored proliferation, migration, and invasion of cancer cells, these results suggested that the methylation of *MAP1LC3v1* might contribute to gastric carcinogenesis. The *MAP1LC3Av1* gene, but not *MAP1LC3B*, was also silenced by methylation in oesophageal squamous cell carcinoma and overexpression of MAP1LC3Av1 in these cells decreased tumorigenesis in vivo [13]. 

#### 2.2.3. Inhibition of DNA Methylation Rescue ATG Gene Expression and Reverse Cancer-Related Phenotypes

A similar methylation-dependent gene silencing was also reported in lung cancer cell lines, but not in lung cancers [15]. Indeed, EGFR-TKI-resistant (epidermal growth factor receptor-tyrosine kinase inhibitor) lung cancer cells presented a decreased methylation of the promoter of *MAP1LC3v1* associated with increased LC3A protein expression [18]. Inhibition of *LC3A* expression using siRNA also decreased LC3B-II levels and cell proliferation, whereas a 5-azadC treatment restored *LC3A* expression in lung cancer PC9 cells and decreased their response to EGFR-TKI. ULK2 expression was also shown to be downregulated following promoter methylation in Glioma cell lines and was restored following a 5-azadC treatment [16]. Moreover, overexpression of ULK2 strongly induced autophagy-mediated cell death in a reactive oxygen species (ROS)-dependent manner. Indeed, treatment of ULK2-overexpressing cells with a ROS scavenger, N-acetyl cysteine (NAC), or an inhibitor of the early steps of autophagy, 3-MA (3-methyladenine), blocked ULK2-induced cell death. Expression of *LAPTM5* gene (lysosomal-associated protein multispanning transmembrane 5) coding a protein associated to lysosomes was downregulated in all (10 out of 10) neuroblastoma tested. *LAPTM5* expression was inversely correlated to promoter methylation and this hypermethylation could be reverted by a 5-azadC treatment [19]. Indeed, *LAPTM5* overexpression provoked a caspase-independent cell death associated with an accumulation of autophagic vesicles. However, since neither wortmannin, an autophagy inhibitor, nor *siATG5* affected LAPTM5-induced cell death, the authors proposed that classical autophagy cell death was not involved. Indeed, LAPTM5 overexpression led to lysosomal destabilization and blocked the autophagy flux, and thus led to cell death [19]. Surprisingly, a hypomethylation of the *ATG4* promoter was frequently associated with the increased expression of the gene and poor prognosis in ovarian carcinoma patients [17].

#### 2.2.4. Indirect Regulation of Autophagy by DNA Methylation in Cancer Cells

The tumor suppressor gene (TSG) *PCDH17* (Protocadherin 17), which is an activator of autophagy, has been shown to be frequently silenced by promoter hypermethylation in gastric and colorectal cancers, but not in normal tissues [20]. Similarly, in hepatocellular carcinoma samples, methylation of the promoter of *BCL2L10* (Bcl-2-like protein 10) was associated with a decreased expression of BCL2L10 [21]. Overexpression of this protein in hepatoma cells restored autophagy in an AMPK-manner and inhibited cell proliferation and tumor growth in mice models. In glioblastoma cells, the TSG *ANKDD1A* (ankyrin repeat and death domain containing 1A) was also frequently silenced by hypermethylation [22]. In normal cells, ANKDD1A interacts with, and activates the expression of *FIH1* (factor inhibiting HIF1) which leads to the downregulation of HIF-1α, and thus to decreased autophagy and induction of cell death. Indeed, activation of the HIF-1-dependent autophagy pathway in GBMs has been previously demonstrated to be a survival pathway.

#### 2.2.5. Molecular Mechanism Controlling Cancer Cell Aggressiveness in a DNA Methylation-Mediated ATG Repression Manner

The treatment of ovarian carcinoma cells with 5-azadC has been shown to induce autophagy and promote cell death [23]. These effects are highly potentiated when 5-azadC is used together with SAHA (suberanilohydroxamic acid), an HDACi. The increase in autophagy in these cells has been demonstrated to be dependent of the induction of expression of the TSG *ARH1* (age-related hearing impairment 1). A treatment of colorectal cancer cells (HT-29) with Se-allylselenocysteine (ASC) has been shown to induce a demethylation of the *PCDH17* promoter associated with an increase in *PCDH17* expression and an activation of autophagy [24]. This effect was described to be linked to the inhibitory effect of ASC on global DNA methylation and DNMT1 expression. Moreover, ASC has been described to significantly decrease HT-29 tumor xenograft growth in mice. It was also recently demonstrated that the activation of CDK4 (cyclin dependent kinase 4), which is frequently observed in cancer cells and linked to the inhibition of senescence, directly phosphorylates DNMT1 and blocks its degradation by autophagy [25]. On the contrary, inhibition of CDKs with palbociclib in prostate cancer cells led to the decrease in DNMT1 levels and overall methylation and contributed to the reduction of the resistance to senescence. Therefore, it has been proposed that the use of CDK inhibitors could be useful in the future to both restore autophagy, inhibit global DNA methylation, and counteract the activation of E2F target genes instead of combining multiple molecules and increasing off-targets [26].

## 3. Histone Deacetylases (HDACs) are Key Regulators of Autophagy

### 3.1. Basics of Histones Acetylation and Deacetylation

Lysine acetylation of histones 3 and 4 are well described epigenetics marks linked to the activation of gene transcription. These modifications are added by histone acetyl transferases (HAT) and removed by histone deacetylases (HDACs). However, it has to be kept in mind that acetylation of nonhistone proteins is also controlled by the same enzymes. To our knowledge, 18 HDACs have been reported in the mammalian genome and these proteins have been divided into the following four classes: class I includes HDAC1, HDAC2, HDAC3, and HDAC8 whichis generally localized in the nucleus; class II is composed of HDACs which shuttle between the cytoplasm and the nucleus and are subdivided into class IIa (HDAC4, HDAC5, HDAC7, and HDAC9) and class IIb (HDAC6 and HDAC10); class III comprises SIRT1 (Sirtuin-1), SIRT2, SIRT3, SIRT4, SIRT5, SIRT6, and SIRT7; and class IV includes HDAC11. Classes I, II, and IV regroup zinc-dependent HDACs, whereas class III contains nicotinamide adenine dinucleotide (NAD+)-dependent HDACs. In cancer cells, acetylation and deacetylation of many promoters has been involved in the specific control of gene expression. Indeed, HDACs have been implicated in different key processes of cancer, including proliferation, inhibition of cell differentiation, cell death, angiogenesis, immune evasion, and autophagy (for a review, [27]). Moreover, HDACis, by modulating these phenotypes, are promising drugs to fight against cancer cells.

### 3.2. HDAC Activities Control Autophagy

The effects of the modulation of HDAC expression or HDAC activity on autophagy has been extensively investigated in cancer cells. Altogether these data, compiled in the Table 2 and detailed below, suggest that HDACs play a key role in the control of autophagy and cancer-related phenotypes associated to autophagy. Moreover, a decrease in acetylation of the lysine 16 of histone 4 (H4K16ac) has been fully correlated to autophagy induction in physiologic conditions and mediated by a loss of HAT hMOF [28]. Indeed, repression of hMOF was dependent of the autophagy process, since blockade of autophagy using BafA1 or 3-MA restores hMOF content. Activation of AMPK, following glucose deprivation, resulted in phosphorylation of acetyl coA synthetase ACSS2 at S659 and modification of its three-dimensional (3D) structure leading to exposition of a NLS domain [29]. P-ACSS2 could interact with importin α5 that promoted importin α/importin β/ACSS2 complex formation, and thus favored ACSS2 nuclear translocation and its further association with TFEB. The ACSS2/TF complex can incorporate acetate from histone acetylation turnover to produce locally acetylcoA and used it to induce H3 acetylation in promoters of ATG-related genes, thus, promoting both autophagy (e.g., activation of lysosome genes *CTSA* (coding cathepsin A), *GBA* (β-glucuronidase), *GUSB* (β-glucosidase), and *LAMP1* (lysosomal membrane protein 1) and cell survival. Indeed, invalidation of ACSS2 expression inhibited cancer cell growth and tumorigenesis in rodent models [30].

Interestingly, acetylation of nonhistone proteins can also control the degradation of specific HDAC targets by autophagy leading to cancer aggressiveness. For example, PCAF (p300/CBP-associated factor) has been shown to favor the acetylation of δ-CATENIN and promote its degradation by autophagy and, consequently, reduce cell growth and mobility [83]. Indeed, overexpression of HDAC1, HDAC4, or HDAC8 markedly increases δ-CATENIN stabilization, to the same levels as the ones observed with autophagy inhibitors (CQ or 3-MA), whereas the inhibition of HDACs (classes I and II) with TSA decreases its stability. In the same way, FOXO1 and 3 transcription factors are effectively described to control gene expression of proteins implicated in autophagosome formation, direct deacetylation of FOXO 1 and 3 by sirtuins induces nuclear localization and increases they ability to bind to DNA [84,85].

### 3.3. HDACi (Classes I and II) Promote Autophagy Cell Death in Cancer Cells

**Figure 2:**
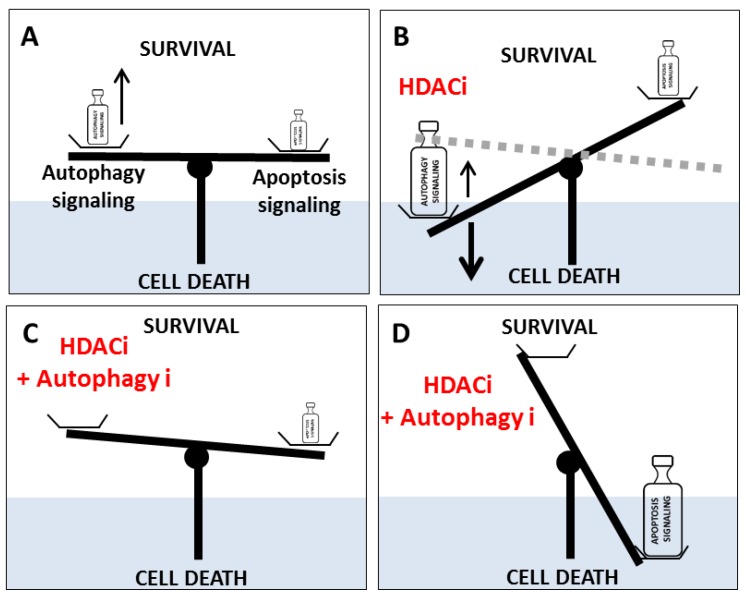
Model of the balance between autophagy and apoptosis governing cell death. (**A**) In cancer cells, the combination of an autophagy-mediated pro-survival signal and weak apoptosis led to cancer cell survival. (**B**) An HDACi can promote a slight increase in apoptosis signaling but also a strong increase in autophagy signaling leading to a balance in favor of autophagy-linked cell death (solid line) or survival (dotted line) depending of both cancer cell model and autophagy induction level. (**C**) The combination of an HDACi and an autophagy inhibitor (autophagy i) in cells resistant to apoptosis, or in the absence of an external signal of apoptosis, led to a balance in favor of survival via the inhibition of autophagy-linked cell death. (**D**) The combination of an HDACi and an autophagy inhibitor (autophagy i) led to a balance in favor of apoptosis.

The inhibition of HDACs, using HDACi, has been associated with an increase of autophagy signaling which could lead to autophagy-linked cell death in many different cancer models (Figure 2). Indeed, SAHA or butyrate promotes both apoptosis and autophagy-induced cell death in HeLa or chondrosarcoma cells (RCS, OUMS-27) [48,56]. Similarly, a molecular mechanism explaining the induction of autophagy by SAHA in breast cancer cells has recently been proposed [60]. Indeed, SAHA has been described to repress the transcription of the autophagy repressor gene SURVIVIN, as well as promote the acetylation of the protein leading to its nuclear translocation. Since nuclear SURVIVIN is less stable than the cytosolic protein and more rapidly degraded, these data might explain how SAHA could favor autophagy. Moreover, inhibition of SURVIVIN expression using *siRNA* in breast cancer cells (MCF-7 and MDA-MB-231) also promoted autophagy, whereas overexpression of SURVIVIN decreased LC3B puncta [60]; *siHDAC2*, *siHDAC3,* and *siHDAC6* also decreased SURVIVIN expression and promoted LC3B-II conversion in these cells, whereas *siHDAC1* led to opposite results [60]. Interestingly, SAHA is known to be more specific towards HDAC3 and HDAC6 than towards HDAC1, and HDAC6 has already been described to be involved in the acetylation of SURVIVIN on K129. Similarly, panobinostat inhibited cell proliferation and promoted both apoptosis and autophagy in Huh7 hepatocellular carcinoma cells [53]. Moreover, depletion of HDAC1 in hepatocellular cancer cells provoked a decrease in cell proliferation and an activation of autophagy-induced cell death [86]. Another HDACi, ZW2-1, also promoted both mitochondria-mediated apoptosis and the number of autophagy vesicles or the levels of LC3B-II in leukemia HL-60 cells [65].

Interestingly, VPA (valproic acid) and SAHA induced the expression of UVRAG in HCT116 colorectal cells and then limited apoptosis in response to 5-FU [87]. Inhibition of autophagy (with CQ or in an *ATG7* knockdown) also enhanced anticancer properties of SAHA, decreased cell proliferation and induction of apoptosis in chronic myeloid leukemia cells, or of vorinostat in colorectal cells in a ROS-dependent manner [88,89]. Indeed, ROS production caused by these compounds led to an accumulation of ubiquitinylated proteins and, then, to increased cell death. Indeed, in U2OS cells, ubiquitination of proteins following SAHA treatment was dependent of the protein HR23B (RAD23 (*S. Cerevisiae*) homolog B) [61] and it has been shown that a decrease of HR23B levels, decreased ubiquitination-mediated apoptosis. All these data, therefore, support that Class I and II HDACi alone can promote autophagy and led to autophagy-linked cell death in a ROS-dependent manner in many different cancer models.

### 3.4. Inhibition of Autophagy Induced by HDACi Blocks Cell Death 

SAHA or OSU-HDAC42 also induced autophagy and autophagy-mediated cell death in hepatocellular carcinoma cells by repressing the AKT/mTOR signaling pathway [5]. However, HDACi-induced cell death could be limited by the addition of the autophagy inhibitor 3-MA or by the inhibition of *ATG5* expression [55]. In Burkitt leukemia and lymphocyte cell lines, VPA also promoted autophagy, and its combination with a mTOR inhibitor, temsirolimus, further increased autophagy, inhibited cell growth, and synergistically promoted autophagy-mediated cell death [67]. Moreover, inhibition of autophagy using 3-MA, BafA1, or *siATG5* decreased growth inhibition mediated by the combined treatment, suggesting that autophagy could act as an antiproliferative pathway in this model. In highly metastatic mice breast cancer cells (4T1), SAHA also increased autophagy (accumulation of LC3B-II and induction of BECLIN1 expression) and potentiated radiation-induced cell death [63]. Indeed, blocking autophagy, using 3-MA, decreased toxicity of the combined treatment. In lung cancer cell lines (PC-9G and H1975), presenting a resistance to EGFR tyrosine kinase inhibitors due to the mutation T790M, SAHA restored the sensitivity to these inhibitors by promoting both apoptosis and autophagy-linked cell death [90]. Inhibition of autophagy by overexpression of spautin-1, which triggers the ubiquitinylation of BECLIN-1, or with *siLC3B* or *siBECN1,* decreased both caspase-dependent and independent apoptosis induced by the combined EGFR tyrosine kinase inhibitor/SAHA treatment in these cells. VPA also potentiated the formation of LC3B-GFP vesicles induced by the multi-kinase inhibitor Pazopanib in sarcoma cell lines. Inhibition of autophagy using *siBECN1* or *siATG5* abrogated VPA/Pazopanib treatment-induced cell death [50]. Romdepsin, another HDACi, also potentiated the response to Bortezomib in gastric carcinoma cells by inhibiting cell proliferation and increasing both apoptosis and autophagy-mediated cell death [91]. Indeed, addition of a ROS scavenger or inhibition of autophagy, using 3-MA, strongly decreased autophagy-induced cell death in these cells (AGS-BDneo and SNU-719) suggesting that the induction of ROS-dependent autophagy was required for the efficiency of the combined treatment [91]. Similarly, AR42, a classI/II HDACi, potentiated cell toxicity of the multi-kinase inhibitor Pazopanib in drug-resistant melanoma cells [92]. Toxicity was mediated by increased death receptor signaling, ER stress signaling and, mainly, autophagy induction (accumulation of BECLIN-1 and positive phosphorylation, S318-P-ATG13, S317-P-ULK1, T172-P-AMK in detriment of negative phosphorylation, S757-P-ULK1, S2448, and S2481-P-mTOR). Moreover, inhibition of autophagy via *siBECN1* or *siATG5* decreased AR42 toxicity. Treatment of T/B-lymphoma cell lines with a combined treatment VPA/doxorubicin increased autophagy (increased LC3B-II and BECLIN-1 levels and autophagy vesicle number) via the activation of AMPK and the inhibition of mTOR, leading to cell toxicity [69]. Inhibition of autophagy in Jurkat cells using drugs (3-MA or BafA1), *siRNA* (*BECN1* or *ATG5*) but not with the caspase inhibitor ZVAD-FMK strongly reduced anti-cell growth effects. Surprisingly, the inhibition of *HDAC1* or *HDAC3* expression, using RNAi, had no effect on the induction of autophagy by VPA/doxorubicin, suggesting that this process was HDAC-independent. Indeed, VPA induced a drop in IP3 levels, and thus a blockade of calcium in mitochondria leading to autophagy, independently of HDAC activity [69]. Similar observations reported that VPA/doxorubicin cotreatment synergistically inhibited cell viability in HepG2 cells, by promoting both apoptosis and autophagy-mediated cell death in a ROS production-dependent manner, cell death which could be reversed by addition of NAC or 3-MA [70]. Pemetrexed/sildenafil (P/S) cotreatment also increased autophagy in non-small cell lung carcinoma (NSCLC) cells (A549) via an induction of CerS6 (ceramide synthase 6) expression and the disruption of HDAC6 content [93]. The inhibition of autophagy by *siBECN1* or *siATG5* decreased P/S-induced toxicity, whereas a cotreatment of P/S with AR42 or VPA increased cell death. Similarly, the combination of VPA with neratinib increased NSCLC cell toxicity by inactivating mTORC1 and mTORC2, and thus inducing both autophagy and cell death [94]. Moreover, inhibition of *BECN1* or *ATG5* expression, or forced expression of an active form of mTOR severely decreased cell death. Cotreatment of uveal melanoma cells with neratinin/MS275 (entinostat) also strongly reduced cell viability by inducing apoptosis and autophagy-linked cell death [95]. Indeed, neratinin/MS275 activated AMPK/ULK1 and ATG13 phosphorylation in a ROS-dependent manner and also severely reduced HDAC6 content leading to cell death. Moreover, overexpression of mTOR significantly reverted cell toxicity mediated by the neratinin/MS275 cotreatment [95]. Indeed, these data strongly suggest that in many cancers, inhibition of autophagy decreased cell death and toxicity mediated by anticancer molecules. As expected, all these data clearly showed, that the inhibition of autophagy blocked autophagy-linked cell death and may favor cancer cell models (Figure 2).

### 3.5. HDACi Treatments Modify the Balance Between Autophagy and Apoptosis

#### 3.5.1. Effects of SAHA and VPA

Although HDACi promote autophagy, which can induce autophagy-mediated cell death (see above), these molecules also act on apoptosis signaling. Indeed, a balance between autophagy and apoptosis has frequently been observed. Since autophagy signaling, following HDACi treatment, can compete with chemical-induced apoptosis for cell survival/death, much data have confirmed that the inhibition of autophagy can promote cell death mediated apoptosis (Figure 2). Indeed, inhibition of autophagy using *siBECN1* in hepatoma cell lines (HepG2, Hep3B, and PLC/PRF/5) enhanced apoptosis induced by the cotreatment SAHA-sorafenib (multi-kinase inhibitor) [96]. Similarly, blockade of panobinostat-induced autophagy using CQ in triple negative breast cancer cells lines (MDA-MB231 and SUM159PT) also led to the accumulation of ubiquitinylated proteins such as SQSTM1/P62-Ub and promoted both cell death in vitro and decrease of tumor growth in xenograph models [54]. In T47D ER (estrogen receptor) positive breast cancer cells, VPA favored apoptosis instead of growth arrest when cells were treated with tamoxifen [97]. However, tamoxifen resistant T47D cells presented a higher autophagy activity, and the inhibition of autophagy in these cells using CQ or *BECN1* silencing restored cell death. In the breast cancer MCF-7 cell line, inhibition of autophagy using 3-MA also potentiated the antiproliferative effect of HDACi, such as CTS203, and promoted apoptosis via the CASPASE 8-mediated cleavage of BECLIN-1 [98]. However, in MCF-7-tamoxifen resistant cells, SAHA alone was sufficient to promote autophagy-dependent cell death [59]. Similar results were obtained in glioblastoma, since the transfection of *siATG7* in T98G cells blocked the induction of SAHA-induced autophagy which was associated to increased LC3 expression and impaired mTOR signaling, and thus favored apoptosis [57]. Combined SAHA/CQ treatments also promoted apoptosis in glioblastoma cell models [99]. Surprisingly, inhibition of the early steps of autophagy using 3-MA, decreased SAHA/CQ-induced cell death suggesting that the accumulation of autophagosomes could be required to induce cell toxicity. These results, therefore, suggest that the benefits of autophagy inhibition against cancer cells are not due to a negative regulation of a pro-survival pathway but rather are dependent on the production of ROS and mitochondria accumulation leading to cell death [99]. Indeed, other data showed that the combination of SAHA with quinacrine, an anti-malaria and autophagy inhibitor, also promoted apoptosis in T-cell leukemia Jurkat cell line by decreasing mitochondrial membrane potential and inhibiting mitophagy [100]. Moreover, a quinacrine/SAHA cotreatment led to ubiquitinylated mitochondrial aggresomes and cell death, effects which could be reverted using the antioxidant NAC.

#### 3.5.2. Others HDACi 

Another HDACi, apicidin, also promoted both apoptosis and autophagy in oral squamous cell carcinoma cells and in mucoepidermoid carcinoma YD-15 cells [45,46,47]. Inhibition of autophagy, using CQ, in these cells further increased cell death linked to the inhibition of the phosphorylation of both AKT and mTOR, the accumulation of LC3B-II, ATG7, and a decrease of SQTM1/P62 levels [45,46,47]. Since apicidin seemed to specifically inhibit HDAC8, a protein frequently overexpressed in oral squamous cell carcinoma tumors, HDAC8 signaling appeared important for the regulation of cell proliferation in this model. Inhibition of HDAC classes I-II in rhabdoid tumor cells, using FK228, also induced autophagy and cell death but the combined inhibition of HDAC and autophagy, with the additional use of CQ, further increased cell death suggesting that autophagy could partially protect these cells from death [66]. Similarly, CQ also increased VPA-induced toxicity in AML cells [101]. A specific inhibition of HDAC8 expression by *siRNA* in oral squamous cell carcinoma cells (YD-10B and FaDu) also promoted both apoptosis and an increase in BECLIN-1, ATG5, ATG12, LC3B-II, and acidic vesicle number, whereas the inhibition of autophagy, using CQ, dramatically increased *siHDAC8*-induced cell toxicity [102]. The inhibitor of HDAC, MGCD0103, promoted apoptosis in primary chronic lymphocytic leukemia (CLL) in detriment of autophagy [50]. Indeed, MGCD0103 activated the PI3K/AKT/mTOR signaling pathway, decreased ATG gene expression (such as *BECN1*, *UVRAG*, *ATG7*, *ATG12*, or *GABARAP*), and thus decreased autophagy flux in primary chronic lymphocytic leukemia, but not in PBMCs (peripheral blood mononuclear cells). However, inhibition of autophagy, using 3-MA or CQ, in primary chronic lymphocytic leukemia inhibited cell growth, whereas combined treatment with MGCD0103 (or VPA) and autophagy inhibitors potentiated cell death [50]. Surprisingly, the blockade of vorinostat-induced autophagy using *shRNA ATG5* or *ATG7*, in Eµ-myc lymphoma cells, had no effect on cell death [62]. These data suggested that, in this model, autophagy induction and cell death mediated by vorinostat were two independent processes.

In 2014, a phase I study tested the combination of SAHA and hydroxychloroquine (HCQ) for the treatment of solid tumors [103]. This study established a maximum daily tolerated dose of 600 mg HCQ and 400 mg SAHA. Among the included patients, one with renal carcinoma presented a stable partial response to treatment. On the basis of the in vitro results, a combination of SAHA with CQ remains promising but efforts are needed to specifically identify the population that could benefit from this treatment. Some additional clinical trials in solid tumors, testing the combination of HDACi and derivative CQ, are currently active or in recruitment (Table 3) (clinicaltrials.gov).

#### 3.5.3. Effect of HDACi/Autophagy and Chemotherapy Combination on Cancer Cells

The inhibition of autophagy, using CQ, severely potentiated cell toxicity mediated by TMZ, SAHA alone, or the combined TMZ/SAHA treatment in glioma cells (C6, U251MG) [58]. Similarly, CQ also increased the effects of SAHA in the glioma GL261 rodent model, suggesting that autophagy induced by SAHA was a protective response against cell death in this type of cancer [58]. 

In Down syndrome-associated myeloid leukemia cells, VPA treatment was associated with a downregulation of 409 autophagy-related genes, associated with an inhibition of autophagy flux and an accumulation of ROS and mitochondria [68]. In MCF-7 cells with acquired resistance to tamoxifen, the HDACi, MHY218, promoted cell arrest and autophagy cell death and suppressed tumor growth in vivo. 

Moreover, cotreatment of TNBC cells (MDA-MB-231 and MDA-MB-468) with LBH549, an HDACi, and Mevastatin, a HMGCR (3-hydroxy-3-methylglutaryl-CoA reductase) inhibitor, increased apoptosis and reduced tumor growth in nude mice models as compared with effects obtained with the molecules used alone [104]. Indeed, this cotreatment induced autophagy by activating the T172-P-AMPK phosphorylation and inhibiting mTOR and P70S6K but at the same time, also inhibited autophagy flux by preventing the formation of the Vsp34/BECLIN-1 complex and decreasing the prenylation of Rab7 (an active form of the small GTPase involved in autophagosome-lysosome fusion) [104]. SAHA and Olaparib, a PARP (poly (ADP-ribose) polymerase 1) inhibitor, cotreatment decreased cell viability in triple negative breast cancer cells (MDA-MB-157, MDA-MB-231, and HCC1143) but not in MDA-MB-468 and HCC70 cells [105]. Indeed, these differences could be explained by the different levels of PTEN (phosphatase and TENsin homolog) in these cells. Moreover, inhibition of PTEN levels using *siRNA* in MDA-MB-231 cells strongly decreased BECLIN-1 and LC3B contents and autophagy vesicle number.

### 3.6. Specific Inhibition of Class II HDAC also Promote Autophagy Cell Death

Since panHDACi seem to promote autophagy-mediated cell death, some studies focused on the role of specific HDACs in this process. Indeed, HDAC4 has been described to deacetylate STAT1 (signal transducer and activator of transcription 1), and thus suppress autophagy in diabetes models [106]. Moreover, in multiple myeloma cells, *miR-29b* specifically targets *HDAC4* mRNA and inhibits HDAC4 expression resulting in the inhibition of cell migration and cell viability and the induction of autophagy [107]. Indeed, treatment of these cells with the pan-HDAC inhibitor, SAHA, promoted *miR29b* expression and blocked pro-cancer HDAC4-associated phenotypes. Interestingly, a combined treatment of a *miR29b* mimics and SAHA synergistically reduced cell aggressiveness in vivo [107]. Inhibition of HDAC5 in HeLa cells using siRNA modulates ROS-related gene expression and provokes an important ROS production leading to apoptotic cell death and a mechanism of elimination of damaged mitochondria by mitophagy. HDAC5 inhibition induces also a metabolic reprogrammation towards glucose and glutamine. Concomitant inhibition of HDAC5 and glutaminase synthase using LMK235 and BTPE, respectively, reduced significantly tumor growth in vivo [108,109]. 

### 3.7. HDAC6 and HDAC10 are Pro-Autophagy Proteins Which Act at the Protein Level

In contrast, HDAC6 is a cytosolic protein favorable to autophagy. HDAC6-/Y mice present lower autophagy than wild type mice [110]. Moreover, HDAC6 can directly interact with HR23B and silencing of HDAC6 in NSCLC A549 cells, using RNAi, promotes both HR23B stabilization and global protein ubiquitination leading to reduced autophagy but increased apoptosis [61]. HDAC6 is also frequently overexpressed in GBMs and high expression of this protein is associated with poor prognosis. Indeed, HDAC6 KO sensitizes the U87 GBM cell line to radiotherapy-mediated autophagic cell death. Indeed, inhibition of autophagy using *siBECN1* or 3-MA restores the formation of colonies in these cells [111]. Nutriment deprivation frequently occurs in solids tumors and its mimics in U87 cells leads to the accumulation of TDP-43 (TAR DNA binding protein), pro-cancer phenotypes and activation of a HDAC6 dependent autophagy at the expense of apoptosis [112]. Indeed, inhibition of HDACs using SAHA or inhibition of autophagic flux using 3-MA or BafA1 in TDP-43 overexpressing cells restores apoptosis. In melanoma cells, AR42 seems to specifically promote HDAC6 degradation by autophagy [113]. However, sulforaphane, a natural HDACi decreases *HDAC6* expression at the mRNA and induces TNBC cell growth arrest and autophagy [64]. Indeed, a decrease of HDAC6 content promotes acetylation and activation of PTEN. PTEN silencing using specific *siRNA* reverses autophagy markers expression, suggesting that sulforaphane-induced autophagy activation is mediated by PTEN [64]. Although little is known concerning the role of HDAC7 on autophagy, its inhibition in mucoepidermoid carcinoma cells, using siRNA also blocks cell proliferation and promotes autophagy (accumulation of LC3B-II and acidic vesicles and decrease of SQSTM1/P62 content) [114].

An increase expression of HDAC10 is associated with autophagy induction and food restriction in rodent models or in Huh7 HCC cells treated with the mTOR inhibitor rapamycin [115]. Moreover, overexpression of HDAC10, in these cells, also promotes autophagy and decreases cell viability. High HDAC10 expression is also associated with *ATGs* gene expression in neuroblastoma but also to poor prognosis [116]. Since CQ addition does not further increase the accumulation of LC3-II caused by HDAC10 depletion in SK-N-BE(2)-C cells, it has been suggested that HDAC10 promotes autophagy and its depletion blocks autophagic flux. Indeed, overexpression of HDAC10 protects NB cells against doxorubicin-mediated toxicity via the acetylation of HSP70. Moreover, cotreatment of SK-N-BE(2)-C cells with doxorubicin and the specific HDAC10 inhibitor bufexamac restores doxorubicin sensitivity and cell death [116].

### 3.8. Ambivalent Role of the SIRT Family (Class III) in Autophagy and Cell Death

In NSCLC (A549 and H1299) cell lines, resveratrol, an activator of SIRT1, increased apoptosis and autophagy (increase of BECLIN-1 and LC3B-II, decrease of SQSTM1/P62, P-MTOR, and P-AKT) [117]. Interestingly, inhibition of SIRT1, using nicotinamide, or blockade of autophagy, using 3-MA, reduced resveratrol-mediated autophagy suggesting that autophagy was mediated by SIRT1 and was a protective event for these cells [117]. Similarly, autophagy-induced by SIRT1 is also involved in 5-FU (5-fluorouracil) resistance in colorectal cancers. Indeed, overexpression of the *lncRNA H19,* a sponge of *miRNA,* or its accumulation following 5-FU treatment in colorectal cancer cells (HCT8 and HCT116) inhibits *miR-194-5p*, a negative regulator of SIRT1, and thus increases SIRT1 expression and autophagy (increase of autophagosomes, LC3B-II, and decrease of SQSTM1/P62) [118]. Moreover, inhibition of autophagy using CQ, abolishes *H19* dependent 5-FU resistance in these cells. SIRT1 also controls the expression of *ATGs*-related genes. Indeed, SIRT1 could deacetylate the RelA/p65 subunit of NFKB (nuclear factor kappa B subunit) at K310, and thus block *BECN1* repression mediated by NFKB [119]. 

In contrast, inhibition of SIRT1 can also promote autophagy. Indeed the blockade of sirtinol-induced autophagy (accumulation of LC3B-II) using 3-MA favors cell toxicity in breast cancer MCF-7 cells [77]. Inhibition of SIRT1 (using siRNA or Ex527 inhibitor) or overexpression of hMOF restores H4K16 acetylation in HeLa cells [28]. However, blockade of autophagy (BafA1, CQ, 3-MA, or ATG7 knock-down) in these cells abrogates rapamycin + Ex524-induced cell death [28]. Inhibition of SIRT1 in endometric cancer cells (Ishikawa) or LumA (MCF-7) cells using MHY2256 also increased apoptosis and accumulated LC3B-II and eventually BECLIN-1, ATG5, ATG7, and autophagy vesicles [79,80]. Treatment of ovarian cancer cells (SKVO3) with different derivatives of a SIRT1 family inhibitors (J11-C1, 15dPGJ2, J19, or MHY2256) affected cell survival and also increased autophagy marker expression (LC3B-II, and/or BECLIN-1, and ATG13), whereas inhibition of autophagy using 3-MA strongly reduced cell death [78,80]. 

In leukemia/lymphoma cells, SIRT2 inhibitors NCO-90/NCO-141 also increased LC3B-II content and apoptosis but neither caspase inhibition nor BafA1 blocked NCO-90/NCO-141-induced cell death suggesting that additional mechanisms were involved [81]. The SIRT6 inhibitor UBCS039 mediated activation of autophagy via the induction of ROS accumulation which by ricochet activated the AMPK-ULK1-mTOR pathway. ROS scavengers prevented UBCS039-mediated autophagy induction. This process required the deacetylase activity of SIRT6 since the H133Y failed to induce autophagy [82].

## 4. HMTs and HDMs also Regulate Autophagy in Cancer Cells

Similar to histone acetylation, histone methylation, which occurs on lysine and arginine, has also been described to be involved in the control of autophagy. Methylation of histones is added by HMTs (histone methyl transferases) and removed by HDMs (histone demethylases). Some specific methylations are associated with active transcription (e.g., H3K4me2/3) but others inhibit gene expression (e.g., H3K27me3). These marks are recognized by epigenetic readers which contribute to modulate gene expression. The effects of the specific inhibition of HMTs or HDMs by epidrugs are summarized in Table 2.

### 4.1. G9a Represses Autophagy Genes Expression in Cancer Cells

The histone methyl transferase G9a has been shown to normally repress *LC3B*, *WIPI*, and *DPTOR* expression by adding the repressive H3K9me2/3 mark on their promoters but this mark has also been shown to be removed following starvation [120]. Chemical inhibition, or the use of a specific *siRNA* targeting *G9a*, led to the induction of autophagy in oral squamous cell carcinoma cells [41,120] or in bone osteosarcoma cells [35]. In breast cancers, *G9a* expression was inversely correlated to *BECN1* expression and patients with high G9a levels and low *BECN1* expression presented the worst prognosis [40]. In MCF-7 breast cancer cells, *BECN1* silencing was correlated to the corecruitment of G9a and DNMT1 on its promoter. In contrast, chemical inhibition of G9a using the compound BIX-01294 promoted *BECN1* expression by inducing the recruitment of NFKB on its promoter in a ROS-dependent manner. Moreover, mono-methylation of H3K9 by G9a was responsible for the activation of the serine-glycine biosynthetic pathway via the induction of the transcription of genes in response to serine deprivation [121]. Indeed, inactivation of G9a led to the depletion of serine and the induction of autophagy-linked cell death. In contrast, a high expression of G9a in cancer patients resulted in poor prognosis and elevated serine levels. In neuroblastoma BE(2)-C cells, BIX-01294 also induced the accumulation of autophagy vesicles and LC3B-II levels, whereas the silencing of G9a, using specific *siRNA*, increased both LC3B-I, LC3B-II, ATG3, ATG7, and ATG12 levels [42]. In glioma cells, BIX-01294 treatment resulted in an AKT-dependent increase of HIF-1α (hypoxia-inducible factor 1) expression, and therefore to increased PKM2 (pyruvate kinase M2) and TIGAR (TP53-induced glycolysis and apoptosis regulator) levels leading to *LC3B* increased expression [122]. In colorectal cancer cells HCT116, BIX-01294 promoted both an accumulation of GFP-LC3B vesicles and LC3B-II levels but also the induction of the transcription of *ATG3*, *ATG4A*, *ATG9A*, and *LC3B* [43]. In AGS gastric cancer cells, the flavonoid kaempferol induced autophagy (accumulation of LC3B-II, BECLIN-1, and ATG5 and decreased levels of SQSTM1/P62) and autophagy-related cell death linked to the decrease in G9a content and the stabilization of IRE1 [44]. Indeed, inhibition of autophagy, using 3-MA, CQ, *siATG5*, or *siLC3B*, restored cell viability in kaempferol-treated cells. ChIP (chromatin immuno-precipitation) experiments also revealed that G9a directly repressed *LC3B* expression by inducing the addition of the repressive mark H3K9me2 on its promoter. In contrast, transfection of *siG9a* restored *LC3B* expression in these cells [44].

### 4.2. Overexpression of EZH2 (Enhancer of Zeste 2 Polycomb Repressive Complex 2 Subunit) in Cancer Cells Repress Autophagy at the Transcriptional Level 

EZH2 has been shown to be recruited onto several autophagy-related promoters (*TSC2*, *RHOA*, *DEPTOR*, *FKBP11*, *RGS16*, and *GPI*) via the chromatin binding protein MTA2 (metastasis associated 1 family, member 2). This recruitment led to the inhibition of their expression via the addition of the repressive mark H3K27me3, and therefore to autophagy blockade [123]. On the one hand, inhibition of EZH2, using GSK343, increased LC3B-II levels and autophagy in U2OS bone osteosarcoma cells [35]. In the MDA-MB-231 breast cancer cells and A549 lung cancer cells, GSK343 treatment also increased LC3B-II levels, as well as the number of autophagy vesicles [37]. On the other hand, *SQSTM1/P62* expression was decreased following GSK343 exposure, whereas 3-MA decreased LC3B-II levels and partially blocked GSK343-induced cell death in these cells. These data supported the idea that EZH2 negatively regulated autophagy flux, and therefore inhibited cell proliferation [37]. Moreover, inhibition of EZH2 by the chemical inhibitor 1o (*N*-((4,6-dimethyl-2-oxo-1,2-dihydropyridin-3-yl)methyl)-5-methyl-1-phenyl-1*H*-pyrazole-4-carboxamide) in neuroblastoma SK-N-BE cells and leukemia K562 cells decreased cell proliferation in an autophagy-dependent manner [38]. In colon cancer cells (HT-29 and HCT-15), inhibition of EZH2, using the compound UNC1999, increased autophagy flux [39]. However, autophagy was more induced when using a combined UNC1999/Gefitinib (EGFRi) treatment which also promoted the inhibition of cell growth and apoptosis. Inhibition of EZH2, using *siRNA* or EZH2i DZNep, in colorectal cancer cells (RKO and HCT116), also increased LC3B-II levels and the expression of the ATG-related protein AMBRA1 (autophagy and BECLIN-1 regulator 1) and favored apoptosis but inhibited cell proliferation and migration [33]. Similar results were obtained in the gastric cancer cells, in which the EZH2 inhibitor GSK126 promoted autophagy by decreasing phosphorylation of mTOR, AKT, and ULK1 leading to LC3B-II accumulation. These effects were further increased when GSK126 was combined with gefitinib and provoked cell death and tumor regression. This effect could be blocked when cells were first treated with the autophagy inhibitor 3-MA [34]. Similarly, in colorectal cancer cells (Lovo, HCT115, and DLD-1), inhibition of EZH2, using the chemical UNC1999 or GSK343 inhibitors, increased autophagy [36], but UNC1999 was still able to promote autophagy in *ATG5^-/-^* cells, cells treated with 3-MA, or cells expressing a dominant negative mutant of ULK1, suggesting that EZH2i could induce non-canonical autophagy in this model. Indeed, autophagy was blocked when DLD-1 cells were first treated with an inhibitor of transcription (actinomycin D) bringing to mind that EZH2i-mediated autophagy was mainly due to the induction of *ATG* gene transcription. Inhibition of LC3B expression using *siRNA* partially rescued cell viability in UNC1999-treated cells [36]. 

### 4.3. miRNA Inhibitors of EZH2 Promote Autophagy and Cell Death

The expression of *miR92b* and EZH2 were inversely correlated in breast cancers and overexpression of *miR92b* favored the accumulation of LC3B-II and GFP-LC3B vesicles and the decrease of P62/SQSTM1 in MCF-7 and MDA-MB-453 cells, during both basal or induced autophagy (starvation or rapamycin treatment) [124]. As expected, inhibition of EZH2 in the same models also led to LC3B-II and autophagy vesicle accumulation. Moreover, overexpression of *miR29b* strongly decreased cell proliferation, migration, and invasion, suggesting that EZH2 negatively controlled autophagy and inhibited its pro-cancer effects [124]. Similarly, the expression of *miR101-3p* was decreased in endometrial carcinoma (EC) as compared with normal adjacent tissues and *miR101-3p* was shown to negatively regulate EZH2 expression [125]. Moreover, both suppression of EZH2 expression and *miR101-3p* increased LC3B-II and BECLIN-1 levels [125]. In laryngeal carcinoma, a low expression of *miR101* was also correlated to a high expression of EZH2. However, in this model, the ectopic expression of *miR101* not only reduced EZH2 expression and cell proliferation but also decreased LC3B-II/LC3B-I levels and accumulated SQSTM1/P62, suggesting that *miR101* could favor or inhibit autophagy depending of the cell model [126]. In glioma, a low expression of *miR340* was associated with a poor prognosis, whereas overexpression of *miR340* strongly decreased *EZH2* expression and inhibited cell proliferation, migration, and invasion [127]. Moreover, forced miR340 expression also decreased phosphorylation of AKT and led to apoptosis, accumulation of LC3B-II, and decreased SQSTM1/P62 contents. In cholangiocarcinoma (CCA) cells (MZChA1, KBMC, and HuCCT1), the specific EZH2 miRNA, *miR124,* was also described to be decreased in tumors as compared with normal tissues [128]. *miR124* or *siEZH2* transfection promoted apoptosis and the accumulation of LC3B-II, BECLIN-1, and LC3B-positive vesicles in these cells. Cotransfection of *siATG5* (or *siBECN-1*) with *siEZH2* (or with *miR124*) inhibited cell death linked to the inhibition of EZH2 [128].

### 4.4. lncRNA Modulators of EZH2 Expression Modulate Autophagy in Cancer Cells at the Transcription and Post-Transcriptional Levels

In chondrosarcoma, both the expression of *EZH2* and its positive regulator *lncRNA HOTAIR* were increased [129]. Inhibition of *lncRNA HOTAIR* reduced cell proliferation, the number of autophagy vesicles and LC3B-II levels in benefit of an activation of apoptosis. Indeed, the *lncRNA HOTAIR* could repress *miR454-3p* expression, in a DNA methylation manner, by inducing the recruitment of the DNMT1/EZH2 complex on the *miR454-3p* promoter. The levels of *miR454-3p* directly affected autophagy, since this *miRNA* can bind to the 3′-UTR of the *ATG12* transcript, and thus induce its degradation [129]. Similarly, the levels of *EZH2* were increased in lung cancers as compared with normal tissues and its levels were correlated to the ones of the *lncRNA MSTO2P,* (a new positive regulator of EZH2 whose mechanism is unknown)*,* data which were coherent since *lncRNA MSTO2P* can positively regulate EZH2 [130]. As expected, the inactivation of *lncRNA MSTO2P* reduced EZH2 expression, as well as cell proliferation and, surprisingly, also autophagy markers (ATG5, LC3B-I, and LC3B-II), suggesting that EZH2 promoted autophagy in this model.

### 4.5. LSD1 (Lysine-Specific Demethylase-1) Negatively Regulates Autophagy at the Protein Level in Cancer Cells

An increase in expression of the histone demethylase LSD1 was frequently observed in cancers. Moreover, a high expression of LSD1 in neuroblastoma was correlated with poor prognosis [74]. Similarly, the expression of LSD1 was also increased in acute myeloid leukemia [72]. In neuroblastoma SH-SY5Y cells, inhibition of LSD1, using chemical inhibitors (TCP or SP2509), blocked LSD1 recruitment onto the *SESN2* (sestrin2) promoter and induced its expression. Since SESN2 is an inhibitor of mTORC1, this led to the restoration of autophagy in these cells [74]. Similarly, inhibition of LSD1 in the U2OS bone osteosarcoma cells, using GSK-LSD1 or 2-PCPA, restored autophagy via the activation of the transcription of *NURP1* and *P62/SQSTM1* manner [35]. However, the inhibition of *ULK1* or *BECN-1* using *siRNA* had no effect on autophagy linked to LSD1-inhibition suggesting that the repression of autophagy mediated by LSD1 mainly occurs at the protein level [35]. In ovarian and endometrial carcinoma cells (ARK2, TOV112D), inhibition of LSD1, using *siRNA* or SP2509, increased ATG7, P62/SQSTM1, and LC3B-II levels and induced GFP-LC3B vesicle formation [75]. Moreover, LSD1 is known to interact with P62/SQSTM1 in the nucleus where it controls its stability in a methylation-independent manner. Inactivation of both P62/SQSTM1 and LSD1 in these cells strongly reduced tumor growth in rodent models. These results strongly supported the idea that the autophagy pathway regulated by LSD1 was independent of BECN-1 and different from the autophagy process induced by starvation. Inhibition of LSD1 in AML cells, using the JL1037 inhibitor, provoked LC3B-II and autophagosome accumulation [72]. Moreover, combination of JL1037 with CQ favored apoptosis in this cell model. In the ovarian carcinoma SKOV3 cells, inhibition of LSD1, using the S2101 inhibitor, activated autophagy by decreasing the phosphorylation of AKT, MTOR, and P70S6K and led to a decrease in P62/SQSTM1 and LC3B-II levels, as well as the number of LC3B-positive vesicles [71]. This effect was accompanied with an increase in apoptosis in these cells. Similarly, inhibition of LSD1, using the NCL1 inhibitor, in the prostate cancer LnCAP cells also promoted apoptosis and induced both LC3B-II levels and autophagosome accumulation [73]. Treatment of LnCAP-injected mice with NCL1 decreased tumor growth. 

Some recent data suggested that autophagy could also be regulated by additional histone demethylases in cancer cells. Indeed, inhibition of KDM6B, using GSKJ4, JMD2, using ML324, or KDM5B, using PBIT, also increased LC3B-II levels and autophagy in U2OS bone osteosarcoma cells [35]. Similarly, in yeasts, Rph1 (KDM4 homolog) negatively regulated autophagy, in a demethylation independent manner, a process that could also be important in mammals to block autophagy in nutrient-rich conditions [131]. 

### 4.6. SKP2-Dependent Repression of CARM1(Coactivator-Associated Arginine Methyltransferase 1) Inhibit Autophagy at the Transcriptional Level in Cancer Cells

Nutriment starvation is a well-known autophagy inducer. Indeed, starvation led to AMPK-dependent phosphorylation of FOXO3a in the nucleus, and consequently to the repression of the SKP2 (S-phase kinase-associated protein 2) [132]. *SKP2* expression was shown to be increased in HCC as compared with normal adjacent tissues and a high expression of SKP2 in cells was correlated to increased proliferation and migration [133]. Since SKP2 is a subunit of an E3 ubiquitin ligase regulating the stability of the nuclear methyltransferase CARM1, starvation-mediated repression of SKP2 resulted in an increase in CARM1 levels and its target, the permissive mark H3R17me2 [132,133]. The concomitant presence of this epigenetic mark with the autophagy transcription factor TFEB on *ATG*-related genes contributed to the activation of autophagy [132]. Indeed, among these targets, transcription of *MAP1LC3B* and *ATG14* genes are directly controlled by this CARM1/TFEB during starvation, a process that could be also involved in cancers when cancer cells are frequently deprived.

## 5. Other Epigenetic Regulators Involved in the Control of Autophagy

### 5.1. The H2Bub1 Mark Positively Regulates Autophagy

A recent, and very elegant, paper revealed the role of the mono-ubiquitination of H2B (H2Bub1) and detailed the multi-epigenetic steps during starvation-induced autophagy [134]. Indeed, the authors showed that starvation in HEK293T cells induced a loss of expression of de novo DNMT3a and DNMT3b, but not of DNMT1, leading to the demethylation of the gene and the expression and activation of the deubiquitinase USP44. This increase in USP44 induced the degradation of H2Bub1 and the decrease in its recruitment onto *ATG*-related genes resulting in their activation. Interestingly, H2Bub1 acted upstream of hMOF since the inhibition of H2Bub1 provoked the inhibition of the acetylation of H4K16, a process associated to induced autophagy, but the inhibition of hMOF failed to modify the levels of H2Bub1.

### 5.2. BRD4 (Bromodomain and Extra-Terminal Domain) Represses Autophagy at the Transcriptional Level

In KP-4 cells, basal autophagy was associated with the repression of *ATG*-related genes mediated by BRD4 (bromodomain-containing protein 4) [31]. Indeed, BRD4 bound acetylated residues and, particularly, the positive mark H4K16ac processed by hMOF and, then, recruited the histone methyltransferase G9a in order to repress the expression of many different autophagy genes (*BECN1*, *VMP1*, *PIK3C3*, *WIPI1*, *ATG2A*, *ATG9B*, *MAP1LC3B*, *SQSTM1*, *OPTN*, *MAP1LC3C*, *TECPR2*, and *SEC24D*) via the addition of the repressive mark H3K9me2 on their promoters [31]. In contrast, the inhibition of BRD4, using specific *siRNAs* or the BET inhibitor JQ1, restored the expression of *ATG*-related genes. Although BET inhibitors are promising drugs for acute myeloid leukemia treatment, leukemia stem cells (LSC) appeared highly resistant, therefore, compromising the efficiency of these new protocols. Indeed, in JQ1-resistant leukemia stem cells, *BECN1* expression, LC3B-II levels, and global autophagy were increased [135]. Chemical inhibition of autophagy, transfection of *siRNA BECN1*, or AMPK inhibition using compound C restored apoptosis in leukemia stem cells treated with JQ1 suggesting that, in these cells, autophagy acted as a pro-survival process which could be counteracted by a combined treatment [135]. 

## 6. Conclusions

**Figure 3:**
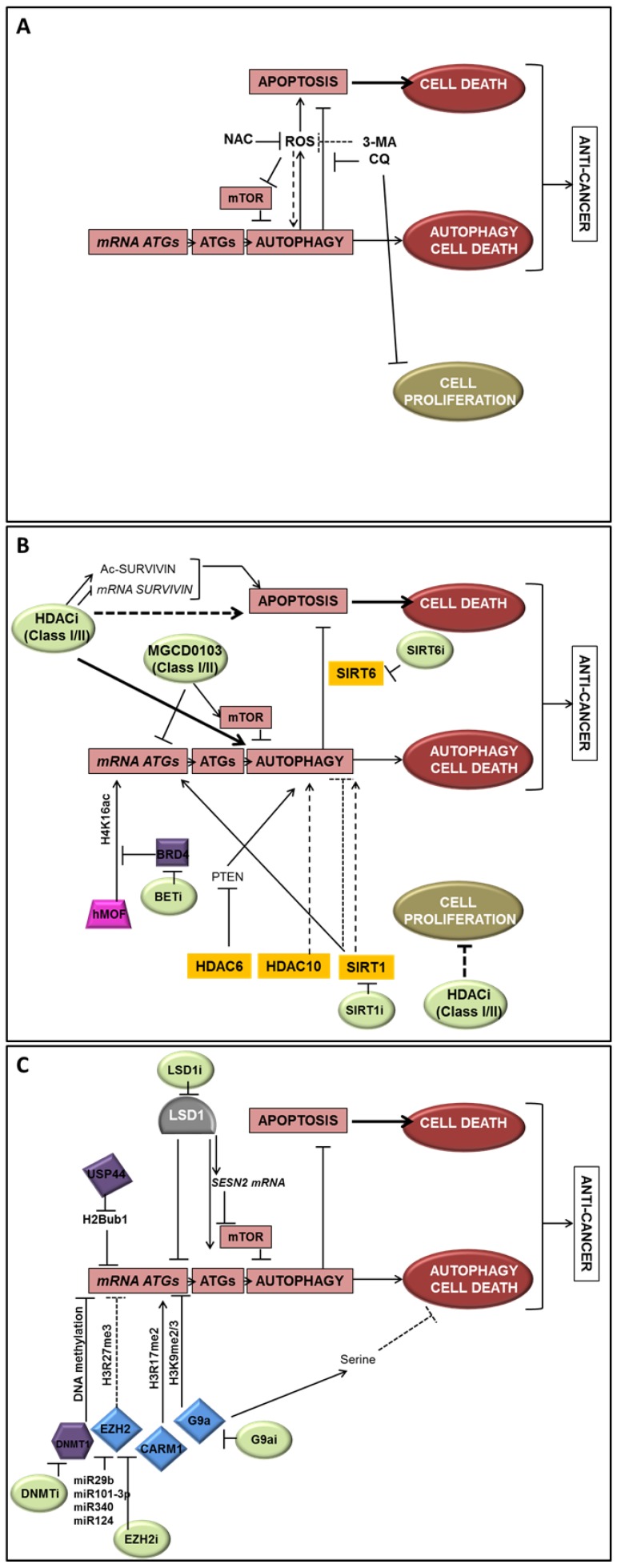
Epigenetic regulation of autophagy in cancer cells. Epigenetic actors and their action on autophagy and cell death are summarized in this figure. A direct transcriptional regulation could be mediated by epigenetic modifiers to promote or inhibit ATG-related genes. An indirect regulation can also be mediated by histones deacetylases and their action on the autophagy process and ROS production. (**A**) Effects of autophagy inducers/inhibitors or ROS on autophagy-linked cell death or survival; (**B**) Effects of HDACi and acetylation/deacetylation signaling on autophagy-linked cell death or survival; (**C**) Effects of histone and DNA methylation regulation and histone ubiquitinylation on autophagy-linked cell death or survival. Inhibitors and miRNA specific of epigenetic modifiers are indicated when their roles have been described in the regulation of autophagy. Arrows represent a positive action on the target whereas bar-headed arrows represent an inhibition. Dotted lines are indicated when the action on the target seems indirect. (clear green: inhibitors; yellow: HDACs; blue: HMTs, pink: HATs; grey: HDMs, purple: other epigenetic proteins).

Although autophagy has been considered for a long time to be mostly regulated at the post-translational level, it is now widely accepted that gene regulation is also determinant for the regulation of autophagy. Moreover, epigenetic modifications, DNA methylation, as well as post-translational histone modifications, events which are also highly involved in cancer promotion and regulation, are more and more linked to autophagy regulation (Figure 3). Indeed, HDACs appeared to repress autophagy signaling while HDACi could improve pro-survival signals mediated by autophagy, as well as autophagy-linked cell death. These data suggested that the inhibition of autophagy in combination with HDACi can be used against tumor cells still capable of apoptosis. Additionally, G9a, EZH2, and LSD1, which are key epigenetic repressors frequently overexpressed in cancer cells and associated with cancer-related phenotypes, have been recently suggested as new potential targets to restore autophagy cell death. Considering the importance of the autophagy-epigenetics links in cancer, it is more than likely that new specific treatments combining autophagy and epigenetic targets will be developed in the near future in order to improve anticancer therapies. 

## Figures and Tables

**Table 1:** Specific promoter methylation of ATG genes in cancers (ND, not determined).

Epigenetic Regulation	Gene	Gene Expression	Cancer	Ref
**DNA hypermethylation**	*ATG2B*	Decreased	Invasive ductal carcinoma	[9]
	*ATG5*	Decreased	Childhood acute lymphatic leukemia	[7]
	*ATG4D*	ND	Invasive ductal carcinoma	[9]
	*ATG9A*	ND	Invasive ductal carcinoma	[9]
	*ATG9B*	Decreased	Invasive ductal carcinoma	[9]
	*ATG16L1*	ND	Medulloblastoma	[14]
	*BECN1*	Decreased	Invasive ductal carcinoma	[8]
	*BNIP3*	Decreased	Colorectal cancer cell lines	[10]
	*GL1*	Decreased	Breast cancer	[11]
	*MAP1LC3*	Decreased	Lung cancer cell lines	[15]
			Childhood acute lymphatic leukemia	[7]
			Gastric carcinoma	[12]
	*ULK2*	ND	Gastric carcinoma	[12]
		Decreased	Glioblastoma	[16]
**DNA hypomethylation**	*ATG4A*	Increased	Ovarian cancer cell lines	[17]

**Table 2:** Described effects of epidrugs on autophagy in cancer cells. Classification from left to right: Epigenetic target, epidrug; cancer cells used in the studies; cancer origin; and effect on autophagy.

Target	Epigenetic Drug	Cells	Cancer Origin	Autophagy	Ref
**BETi**	JQ1	KP-4	Pancreas carcinoma	Increase ATG gene expression	[31]
**DNMT**	5-aza-dC	Hey	Ovarian carcinoma	Increase of AVOs	[23]
		K-562	Chronic myeloid leukemia	Increase of LC3B-II	[32]
**EZH2**	DZNep	RKO, HCT116	Colorectal carcinoma	Increase of LC3B-II	[33]
	GSK126	MG803	Gastric carcinoma	Increase of LC3B-II and decrease of phospo AKT/mTOR/ULK1	[34]
	GSK343	U2OS	Bone carcinoma	Increase of LCB-II, decrease of P62/SQSTM1	[35]
		HCT115, DLD-1	Colorectal carcinoma	Increase of LC3B flux and autophagic vesicles	[36]
		MDA-MB-231	Triple negative breast cancer	Increase of LC3B and SQSTM1/P62 fluxes	[37]
	1o	K562	Myelogenous leukemia	Increase of LCB-II	[38]
		SK-N-BE	Neuroblastoma	Increase of LCB-II	[38]
	UNC1999	HT-29, HC-T15	Colon cancer	Increase LC3B-II flux	[39]
		Lovo HCT115, DLD-1	Colorectal carcinoma	Increase LC3B-II flux and autophagic vesicles	[36]
**G9a**	BIX01294	U2OS	Bone carcinoma	Increase of LC3B-II and vesicles	[35]
		HeLa	Cervix carcinoma	Increase of LC3B-II and vesicles	
		MCF-7	Breast (LumA)	Increase of BECLIN-1	[40]
		TCA8113	Tongue squamous cell carcinoma	Increase of LC3B-II	[41]
		BE(2)-C, SHEP1	Neuroblastoma	Increase of vesicles and LC3B-II	[42]
		HCT116	Colorectal carcinoma	Increase of vesicles and LC3B-II, activation of ATG gene expression	[43]
	Kaempferol	AGS	Gastric carcinoma	Increase of LCB-II, decrease of P62/SQSTM1, autophagic cell death	[44]
**HDAC**	Apicidin	YD-8,YD-10B, AT84	Oral squamous carcinoma	Increase of LC3B-II, ATG5 and AVOs	[45,46]
		YD-15	Mucoepidermoid carcinoma	Increase of LC3B-II and AVOs, decreased P62	[47]
	Butyrate	HeLa	Cervix carcinoma	Increase of vesicles and autophagic cell death	[48]
	ITF2357 (givinostat)	U87, U251	Glioblastoma	Increase of LCB-II, ATG5, ATG7, BECLIN-1, autophagic cell death	[49]
	MGCD0103	Primary B-cell chronic lymphocytic	B-cell chronic lymphocytic leukemia	decrease of LCB-II, ATG5, ATG12, P62/SQSTM1, BECLIN-1 and of autophagic flux	[50]
	MHY218	MCF-7	Breast (LumA)	Increase of LCB-II and BECLIN-1	[51]
	Panobinostat (LBH589)	L428, L540	Hodgkin lymphoma	Increase of LCB-II and vesicles	[52]
		Huh7	Hepatocarcinoma	Increase of LCB-II and decrease of P62/SQSTM1	[53]
		MDA-MB-231, SUM159PT	Triple negative breast cancer	Increase of LCB-II, BECLIN-1 and decrease P62/SQSTM1	[54]
	OSU-HDAC42	HCCs	Hepatocarcinoma	Increase of LCB-II and vesicles	[55]
	SAHA (Vorinostat)	HeLa	Cervix carcinoma	Increase of vesicles	[48]
		RCS, OUMS-27	Chondrosarcoma	Increase of LCB-II and vesicles	[56]
		HCCs	Hepatocarcinoma	Increase of LCB-II and vesicles	[55]
		T98G, U251MG, C6	Glioblastoma	Increase of LCB-II, vesicles and AVOs	[57,58]
		MCF-7, MCF-7 Tamox-R	Breast (LumA)	Increase of LCB-II, BECLIN-1, vesicles, AVOs, autophagic cell death and decrease of P62/SQSTM1	[59,60]
		MDA-MB-231	Triple negative breast cancer	Increase of LCB-II, BECLIN-1 and AVOs and decrease of P62/SQSTM1	[60]
		HUT78	T-cell lymphoma	Increase of LCB-II	[61]
		MYLA	Cutaneous T-cell lymphomas	Increase of LCB-II	[61]
		A2058, A375	melanoma	Increase of LCB-II	[61]
		HCT116, HCT15	Colorectal carcinoma	Increase of LCB-II, ATG5	[61]
		µ-myc	B-cell lymphoma	Increase of LCB-II	[62]
		4T1	Breast cancer	Increase of vesicles	[63]
	Sulforaphane	MDA-MB-231, BT549, MDA-MB-468	Triple negative breast cancer	Increase of LCB-II, BECLIN-1 and vesicles and decrease of P62/SQSTM1	[64]
	ZW2-1	HL-60	Leukemia	Increase of LCB-II, vesicles	[65]
**HDAC (class I/II)**	FK228	KP-MRT-NS	Malignant rhabdoid tumors	Increase of LC3B-II and vesicles, autophagic cell death	[66]
	Valproate	A2058, A375	Melanoma	Increase of LCB-II	[61]
		Namalwa, Raji, Daudi, Ramos	Burkitt leukemia/lymphoma	Increase of LCB-II, BECLIN-1, vesicles and decrease of P62/SQSTM1, P-MTOR	[67]
		CMK	Acute megakaryocytic leukemia	Decrease of *ATG7* mRNA and increase of GFP-LC3B vesicles	[68]
		Jurkat, H9	T-lymphoma	Increase of LCB-II, vesicles and decrease of P-MTOR	[69]
		SU-DHL-4	B-lymphoma	Increase of LCB-II, vesicles and decrease of P-MTOR	[69]
		HepG2	Hepatocarcinoma	Increase of LCB-II and AVOs	[70]
		HeLa	Cervix carcinoma	Increase of LC3B vesicles	[28]
**JMJD2**	ML324	U2OS	Bone carcinoma	Increase of LCB-II	[35]
**KDM5B**	PBIT	U2OS	Bone carcinoma	Increase of LCB-II and P62/SQSTM1	[35]
**KDM6B/JMJD3**	GSKJ4	U2OS	Bone carcinoma	Increase of LCB-II, decrease of P62/SQSTM1	[35]
**LSD1**	GSK-LSD1	U2OS	Bone carcinoma	Increase of LCB-II, decrease of P62/SQSTM1	[35]
	S2101	SKOV3	Ovarian carcinoma	Increase of LCB-II, GFP-LC3B vesicles and decrease P62/SQSTM1	[71]
	JL1037	THP-1, Kasumi-1	Acute myeloid leukemia	Increase of LCB-II and vesicles	[72]
	NCL1	LnCAP	Prostate carcinoma	Increase of LCB-II flux, vesicles	[73]
	SP2509	SHSY5Y	Neuroblastoma	Increase of LCB-II	[74]
		ARK2, TOV112D	Ovarian carcinoma	Increase of LCB-II, ATG7 and P62/SQSTM1	[75]
	TCP	SHSY5Y	Neuroblastoma	Increase of LCB-II and GFP-LC3B vesicles	[74]
		HO8910	Ovarian carcinoma	Increase of LCB-II	[76]
**SIRT1**	Sirtinol	MCF-7	Breast (LumA)	Increase of LCB-II and AVOs	[77]
	J11-C1	SKOV3	Ovarian carcinoma	Increase of LCB-II and BECLIN-1	[78]
	15dPGJ2	SKOV3	Ovarian carcinoma	Increase of LCB-II	[78]
	J19	SKOV3	Ovarian carcinoma	Increase of LCB-II, ATG3 and BECLIN-1	[78]
	MHY2256	Ishikawa	Endometric carcinoma	Increase of LCB-II, ATG5/7, BECLIN-1, AVOs	[79]
		SKVO3	Breast (LumA)	Increase of LCB-II and AVOs	[80]
		MCF-7	Ovarian carcinoma	Increase of LCB-II and AVOs	[80]
	NCO-90/NCO141	HL60, MT2, SIT1, Jurkat	Leukemia/T-lymphoma	Increase of LCB-II	[81]
**SIRT6**	UBCS039	H1299	Non-small cell lung carcinoma	Increase of LCB-II, GFP-LC3B vesicles and autophagic flux	[82]
		HeLa	Cervix carcinoma	Increase of LCB-II, GFP-LC3B vesicles and autophagic flux	[82]

**Table 3:** Clinical trials testing autophagy inhibitor and HDACi.

Trial Reference	Study	Cancer	Drugs	Status
NCT01023737	Hydroxychloroquine + vorinostat in advanced solid tumors	Malignant solid tumor	Hydroxychloroquine Vorinostat	Active, not recruiting
NCT01266057	Sirolimus or vorinostat and hydroxychloroquine in advanced cancer	Advanced cancers	Hydroxychloroquine Sirolimus Vorinostat	Active, not recruiting
NCT03243461	International cooperative phase III trial of the HIT-HGG study group (HIT-HGG-2013)	Glioblastoma WHO Grade IV Diffuse Midline Glioma Histone 3 K27M WHO Grade IV Anaplastic Astrocytoma WHO Grade III	Temozolomide + Valproic Acid Temozolomide + Chloroquine	Recruiting
NCT02316340	Vorinostat Plus Hydroxychloroquine versus regorafenib in colorectal cancer	Colorectal cancer	Vorinostat Hydroxychloroquine Regorafenib	Active, not recruiting

## Abbreviations

**ACSS2** (Acyl-CoA Synthetase-2), **AKT** (AKT Serine/Threonine Kinase 1), **AMBRA1** (Autophagy And Beclin 1 Regulator 1), **AMPK** (AMP-activated protein kinase), **ASC** (Se-allylselenocysteine), **ATG** (autophagy-related), **ATG4** (Autophagy-related 4), **AVO** (acidic vesicular organelles), **5-azadC** (5-aza-deoxycytidine), **BafA1** (bafilomycin-1), **BET** (Bromodomain and extraterminal domain) inhibitors, **BMI1** (BMI1 Proto-Oncogene, Polycomb Ring Finger), **BCL2L10** (Bcl-2-Like Protein 10), **BNIP3** (BCL2/adenovirus E1B 19 kDa protein-interacting protein 3), **CARM1** (coactivator-associated arginine methyltransferase 1), **CerS6** (Ceramide Synthase 6), **ChIP** (chromatin immune-precipitation), **CQ** (chloroquine), **CTSA** (coding cathepsin A), **DNMT** (DNA methyl transferase), **E2F** (E2F Transcription Factor), **EGFR** (Epidermal Growth Factor Receptor), **ER** (estrogen receptor), **EZH2** (enhancer of zeste 2 polycomb repressive complex 2 subunit), **GBA** (β-glucuronidase), **GUSB** (β-glucosidase), **HDAC** (histone deacetylase), **HDM** (histone demethylase), **HMGCR** (3-Hydroxy-3-Methylglutaryl-CoA Reductase), **HMT** (histone methyltransferase) **HR23B** (RAD23 (S. Cerevisiae) Homolog B), **FIH1** (Factor Inhibiting HIF1), **5-FU** (5-Fluorouracil), **GL1** (GABARAPL1, GABA Type A Receptor Associated Protein Like 1), **H3** (Histone 3), **HCC** (hepatocellular carcinoma), **HCQ** (hydroxychloroquine), **HIF-1α** (hypoxia-inducible factor 1), **JMD** (Jumonji Domain Containing), **KDM** (Lysine Demethylase), **LAMP1** (lysosomal membrane protein 1), **LAPTM5** gene (Lysosomal-associated protein multispanning transmembrane 5), (loss of heterozygosity), **LSD1** (Lysine-specific demethylase-1), **3-MA** (3-methyl-adenine), **MAP1LC3v1** (Microtubule Associated Protein 1 Light Chain 3 Alpha), **MTOR** (Mechanistic Target Of Rapamycin), **NAC** (N-acetyl cysteine), **NFKB** (Nuclear Factor Kappa B Subunit), **NSCLC** (non-small cell lung carcinoma), **PARP** (Poly(ADP-Ribose) Polymerase 1), **PCAF** (p300/CBP-associated factor), **PCDH17** (Protocadherin 17), **PKM2** (Pyruvate kinase M2), **PTEN** (Phosphatase And Tensin Homolog), **ROS** (Reactive Oxygen Species), **SAHA** (suberanilohydroxamic acid), **SIRT** (Sirtuin), **SP1** (Sp1 Transcription Factor), **SQSTM1** (Sequestosome 1), **STAT1** (Signal Transducer And Activator Of Transcription 1), **TDP-43** (TAR DNA Binding Protein), **TIGAR** (TP53-induced glycolysis and apoptosis regulator), **TKI** (Tyrosine kinase inhibitor), **TNBC** (triple negative BC), **TSA** (trichostatin A), **TSS** (transcriptional start site), **TSG** (tumor suppressor genes), **USP44** (Ubiquitin Specific Peptidase 44), **ULK** (Unc-51 Like Autophagy Activating Kinase), **UVRAG** (ultraviolet irradiation resistance-associated), **VPA** (valproic acid).
